# Immunostimulatory Activity of Protein Hydrolysate from Oviductus Ranae on Macrophage *In Vitro*

**DOI:** 10.1155/2014/180234

**Published:** 2014-12-22

**Authors:** Di Huang, Lubing Yang, Chenlu Wang, Sihui Ma, Li Cui, Shiyang Huang, Xia Sheng, Qiang Weng, Meiyu Xu

**Affiliations:** ^1^College of Biological Science and Technology, Beijing Forestry University, Beijing 100083, China; ^2^Beijing Key Laboratory of Forest Food Processing and Safety, Beijing Forestry University, Beijing 100083, China

## Abstract

Oviductus Ranae is the dry oviduct of *Rana chensinensis*, which is also called *R. chensinensis* oil. Oviductus Ranae is a valuable Chinese crude drug and is recorded in the Pharmacopoeia of the People's Republic of China. The aim of this study was to investigate the immunostimulatory activity of protein hydrolysate of Oviductus Ranae (ORPH) and to assess its possible mechanism. Immunomodulatory activity of ORPH was examined in murine macrophage RAW 264.7 cells. The effect of ORPH on the phagocytic activity of macrophages was determined by the neutral red uptake assay. After treatment with ORPH, NO production levels in the culture supernatant were investigated by Griess assay. The mRNA and protein expressions of inducible nitric oxide synthase (iNOS) were detected by RT-PCR and Western blotting. The production of TNF-*α*, IL-1*β*, and IL-6 after treatment with ORPH was measured using ELISA assay. In addition, NF-*κ*B levels were also investigated by Western blot. The results showed that ORPH enhanced the phagocytosis of macrophage, increased productions of TNF-*α*, IL-1*β*, IL-6, and NO in RAW 264.7 cells, and upregulated the mRNA and protein expression of iNOS. Besides, NF-*κ*B, levels in RAW 264.7 cells were elevated after ORPH treatment. These findings suggested that ORPH might stimulate macrophage activities by activating the NF-*κ*B pathway.

## 1. Introduction

Macrophages are a type of differentiated tissue cells that originate as blood monocytes. The cells have several functions such as the removal of cell debris, the killing of pathogenic microorganisms, and the processing and presentation of antigens to lymphocytes [1, 2]. Macrophages are the first cells to recognize invading foreign bodies and are central to cell mediated and humoral immunity. Therefore, the activation of macrophages is a key event for effective innate and adaptive immunity. It is reported that activated macrophages can defend against pathogen invasion by secreting proinflammatory cytokines and releasing some inflammatory molecules such as tumor necrosis factor- (TNF-) *α*, interleukin- (IL-) 1*β*, interleukin- (IL-) 6, or nitric oxide (NO) [3]. NO, as a free-radical gas, is synthesized by inducible nitric oxide synthase (iNOS) and mediates diverse functions, including vasodilatation, neurotransmission, immunoresponses, and inhibition of platelet aggregation and of extracellular matrix production [4, 5]. NO has been identified as the major effector molecule involved in the destruction of tumor cells by activated macrophages [6]. Moreover, the involvement of NO during nonspecific host defense, macrophage-mediated killing, and the inhibition of the proliferation of microorganisms and tumor cells both* in vitro* and* in vivo* has previously been demonstrated [7].

Nuclear factor *κ*B (NF-*κ*B) is a transcriptional factor that regulates a battery of genes that are critical to innate and adaptive immunity, cell proliferation, inflammation, and tumor development. In macrophages, NF-*κ*B, in cooperation with other transcription factors, coordinates the expression of the genes encoding TNF-*α*, IL-1*β*, IL-6, and IL-8 [8]. Moreover, NF-*κ*B plays a critical role in the activation of immune cells by upregulating the expression of many cytokines essential for the immune response [9]. NF-*κ*B activation also results in the upregulation of antiapoptotic genes thereby providing cell survival mechanism to withstand the physiological stress that triggered the inflammatory response. Enhanced NF-*κ*B activity can be directly induced by mutations of NF-*κ*B genes and/or oncogenes that activate the NF-*κ*B signaling pathway.

The Chinese brown frog (*Rana chensinensis*) is a special amphibian in northeastern China and has been used widely in Traditional Chinese Medicine [10]. However, one specific physiological phenomenon of* R. chensinensis* is that its oviduct expands prior to hibernation, instead of during the breeding period [11]. Moreover, the dessicated product of expanded oviduct of the female* R. chensinensis*, Oviductus Ranae, is a valuable Chinese crude drug and is recorded in the Pharmacopoeia of the People's Republic of China [12]. Oviductus Ranae is one of the best-known and highly valued oriental foods and medicines. Previous studies have shown that Oviductus Ranae is mainly composed of proteins, the content of which are up to 50% or more [13]. Traditional Chinese Medicine holds that Oviductus Ranae can nourish yin, moisten lung, and replenish the kidney essence. Meanwhile, activities of Oviductus Ranae such as antiaging, antilipemic, antioxidation, and antifatigue have also been demonstrated by modern pharmacological studies [14]. Although Oviductus Ranae was reported to augment the immune response by modulating macrophage function [15], the precise mechanism of its augmentation of cell-mediated immunity remains to be elucidated. In this study, we for the first time set out to explore the effect of protein hydrolysate of Oviductus Ranae on macrophage function, which could be one of major mechanism underlying Oviductus Ranae's immunomodulatory effect. Our results strongly indicate that protein hydrolysate of Oviductus Ranae enhances some of the key macrophage physiological parameters* in vitro*, including phagocytosis, NO secretion, and production of TNF-*α*, IL-1*β*, and IL-6.

## 2. Materials and Methods

### 2.1. Materials

Oviducts Ranae were collected from the adult female Chinese brown frogs (obtained during the prehibernation from Jilin Baekdu Mountain Chinese Brown Frog Breeding Farm, Jilin Province, China). All the procedures on animals were carried out in accordance with the Policy on the Care and Use of Animals by the Ethical Committee, Beijing Forestry University. Murine macrophage cell line RAW 264.7 (ATCC number TIB-71) and cell culture materials were purchased from the Cell Culture Centre of the Institute of Basic Medical Sciences, Chinese Academy of Medical Sciences, Beijing, China. Papain, trypsin, pepsin, neutral protease, and alkaline protease were purchased from Sigma. Griess reagent and nuclear (or cytoplasmic) protein extraction kit were obtained from Beyotime Biotechnology, China. Antibody against iNOS was from Santa Cruz Biotechnology Inc. (Dallas, TEX, USA). ELISA kits (for TNF-*α*, IL-1*β*, and IL-6) were purchased from Cusabio Biotech, China. Anti-NF-*κ*Bp65 and anti-I*κ*B-*α* were obtained from Biosynthesis Biotechnology, China.

### 2.2. Oviductus Ranae Protein and Protein Hydrolysate Preparation

Frozen Oviductus Ranae was thawed, chopped, and immediately mixed with phosphate buffered saline (PBS, 0.1 M pH 7.4) at 1 : 10 Oviductus Ranae to buffer ratio by continuous stirring combined with ultrasonic treatment for 2 h. The mixture was centrifuged and the supernatant was collected. Protein was extracted from the supernatant by ammonium sulfate precipitation technique. Oviductus Ranae protein isolate was dispersed in distilled water obtain 6.0% (w/v) protein slurry. Various enzymes (papain, trypsin, neutral protease, pepsin, and alkaline protease) were added to the protein solution, respectively, at varying temperature and pH as shown in Table 1. The temperature and pH of the slurry were maintained constant for 3 h, after which the hydrolysis was stopped by heating the slurry to 95°C and held for 10 min. The hydrolysates were cooled to room temperature and centrifuged at 3,000 g for 10 min. The clear supernatant was collected and freeze-dried and stored at −20°C as protein hydrolysate of Oviductus Ranae.

### 2.3. Cell Culture

The RAW 264.7 mouse macrophage cell line was cultured in DMEM supplemented with 10% fetal bovine serum, 2 mM L-glutamine, 100 U/mL penicillin, and 100 *μ*g/mL streptomycin at 37°C in a 5% humidified incubator with 5% CO_2_.

### 2.4. Cytotoxicity Assay on Macrophages

Cytotoxicity on RAW 264.7 cells was measured by conventional MTT assay. Cells were cultured in 96-well plates at a density of 3 × 10^4^ cells/mL with test substances for 48 h at 37°C and 5% CO_2_. After incubation, 20 *μ*L of MTT (3-(4,5-dimethylthiazol-2-yl)-2,5-diphenyltetrazolium bromide, 5 mg/mL) reagent was added to each well and incubated for 4 h at 37°C in the dark. The culture medium containing the MTT solution was replaced by 200 *μ*L DMSO (dimethyl sulfoxide) and shaken in the dark for 15 min at room temperature for complete dissolution of the MTT formazan productions. The optical density of each well was measured by absorbance at 570 nm using a Benchmark Plus microplate reader (Bio-Rad).

### 2.5. Neutral Red Uptake Assay for Macrophage Phagocytosis

The phagocytic ability of macrophage was measured by neutral red uptake assay [16, 17]. After cells were cultured with test substances for 48 h, 100 *μ*L neutral red solutions (dissolved in 10 mM PBS with the concentration of 0.075%) was added and incubated for 1 h. The supernatant was discarded and the cells in 96-well plates were washed with PBS twice to remove the neutral red that was not phagocytized by RAW 264.7 cells. Then, cell lysate (ethanol and 0.01% acetic acid at the ratio of 1 : 1, 100 *μ*L/well) was added to lyse cells. After cells were incubated in room temperature overnight, the optical density at 540 nm was measured by a microplate reader.

### 2.6. Nitric Oxide (NO) Production by Macrophages

Measurement of nitrite in medium was used as an indicator of NO production [9, 16]. RAW 264.7 cells (5 × 10^5^ cells/mL) were cultured in 96-well plates with test substances. After 24 h, culture supernatants were collected and nitrite, the stable reaction product of NO with molecular oxygen, was measured using Griess reagent. Equal volumes of Griess reagent and sample were incubated together at room temperature for 10 min. Nitrite production was determined by comparing the absorbance at 540 nm with a standard curve generated by NaNO_2_.

### 2.7. RT-PCR Analysis of iNOS mRNA

Total RNA from 2 × 10^6^ cells was extracted with 1 mL Trizol reagent. The RNA precipitate was suspended in 10 *μ*L of DEPC-treated water. The reaction tubes for cDNA synthesis contained 2 *μ*g total RNA, 10 *μ*M oligo (dT) 18 primer, 2.5 mM dNTPs, 25 U RNase inhibitor, and 200 U Moloney murine leukemia virus reverse transcriptase in total 25 *μ*L volume per tube with the reaction of: 5 min at 75°C, 1 h at 37°C, and 5 min at 95°C. The cDNA was stored at −20°C before used. PCR amplification of cDNA encoding iNOS was carried out in 25 *μ*L reaction volume containing 1 *μ*L cDNA template, 2.5 *μ*L of 10X Taq DNA polymerase buffer, 1.5 *μ*L MgCl_2_ (25 mM), 0.5 *μ*L dNTP mix (10 mM), 1 *μ*L each of primer (10 *μ*M), and 1 U Taq DNA polymerase. The sense and antisense primers for iNOS were 5′-GTCTTGCAAGCTGATGGTCA-3′ and 5′-GGCCTCAGCTTCTCATTC-TG-3′, respectively. The expected product length is 602 bp. As for *β*-actin, the sense and antisense primers were 5′-AGGCATCCTGACCCTGAAGTAC-3′ and 5′-TTCATGAGGTAGTCTGTCAG-3′, respectively. The length of expected product is 389 bp. The reaction mixture was denatured at 94°C for 5 min and subjected to 30 cycles of 30 s at 94°C, 50 s at 52°C, 50 s at 72°C, and a final extension step of 10 min at 72°C. The amplification was accomplished in 2720 Thermal Cycler (Gene Co. Ltd.). The PCR products were analyzed on a 2% agarose gel containing ethidium bromide. The bands of PCR products were visualized under UV light and analyzed with iPP 6.0. The results were expressed with the relative intensity compared with *β*-actin.

### 2.8. Western Blot

Proteins Expression of iNOS, NF-*κ*B, and I*κ*B-*α* was measured by Western blot analysis. RAW 264.7 cells were cultured in 6-well plates with test substances for 24 h [9, 18]. Cells were collected by 0.02% EDTA kept on ice for 30 min, before the nuclear proteins and cytoplasmic proteins were isolated, respectively, by using nuclear and cytoplasmic protein extraction kit. Protein was then resolved by 10% SDS-PAGE and transferred onto polyvinylidene difluoride membranes. After blocking, the membranes were incubated with the target antibody. The horseradish peroxidase-conjugated secondary antibody to IgG was used. *β*-actin was selected as the endogenous control.

### 2.9. Measurement of Cytokine Production

For cytokine immunoassays, RAW 264.7 cells were cultured for 24 h at a density of 3 × 10^5^ cells/mL in 96-well plates. Supernatants were removed at the indicated time, and TNF-*α*, IL-1*β*, and IL-6 production were quantified by sandwich immunoassays using the protocol supplied by ELISA kits.

### 2.10. Preparation of Peptide Fractions

To obtain peptide fractions with different molecular weights, the hydrolysates were subjected to ultrafiltration using 3, 5, and 10 kDa molecular weight cut-off membranes. Three fractions, namely, <3 kDa, 3 kDa–5 kDa, and 5 kDa–10 kDa, were obtained. All fractions were lyophilized for use in the experiments.

### 2.11. Statistical Analysis

The data were expressed as means ± S.D. The significance of difference was evaluated with one-way ANOVA, followed by Student's *t*-test to statistically identify differences between the control and treated groups. Significant differences were set at *P* < 0.05 and *P* < 0.01.

## 3. Results

### 3.1. Effect of Protein Hydrolysate of Oviductus Ranae on Cell Viability

**Figure 1:**
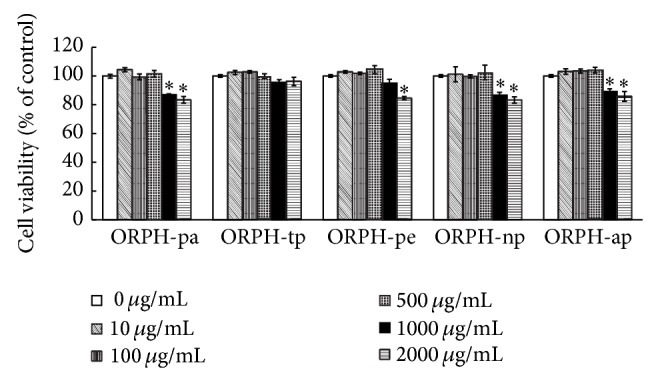
Effect of protein hydrolysate of Oviductus Ranae on cell viability. RAW 264.7 cells were pretreated with test substances (0, 10, 100, 500, 1000, and 2000 *μ*g/mL) for 48 h and were used to test the cytotoxicity by MTT assay. OR, Oviductus Ranae; ORP, Oviductus Ranae protein; ORPH-pa, Oviductus Ranae protein hydrolysate prepared with papain; ORPH-tp, Oviductus Ranae protein hydrolysate prepared with trypsin; ORPH-pe, Oviductus Ranae protein hydrolysate prepared with pepsin; ORPH-np, Oviductus Ranae protein hydrolysate prepared with neutral protease; ORPH-ap, Oviductus Ranae protein hydrolysate prepared with alkaline protease. Control, untreated cells.

Various protein hydrolysates of Oviductus Ranae were prepared by papain, trypsin, pepsin, neutral protease, and alkaline protease (referred to as ORPH-pa, ORPH-tp, ORPH-pe, ORPH-np, and ORPH-ap, resp.). To make sure the measurement of phagocytosis and NO production can well represent the cell function without any changes in cell quantity, it was necessary to evaluate the cytotoxic effect of protein hydrolysates of Oviductus Ranae on RAW 264.7 cells before further tests were carried out. MTT assay indicated that the protein hydrolysates did not show cytotoxic effects on RAW 264.7 cells in concentration ranging from 0 to 500 *μ*g/mL (Figure 1). Thereafter, the cells were treated with the hydrolysates in 500 *μ*g/mL concentration during subsequent experiments.

### 3.2. Effect of Protein Hydrolysate of Oviductus Ranae on Phagocytosis

**Figure 2:**
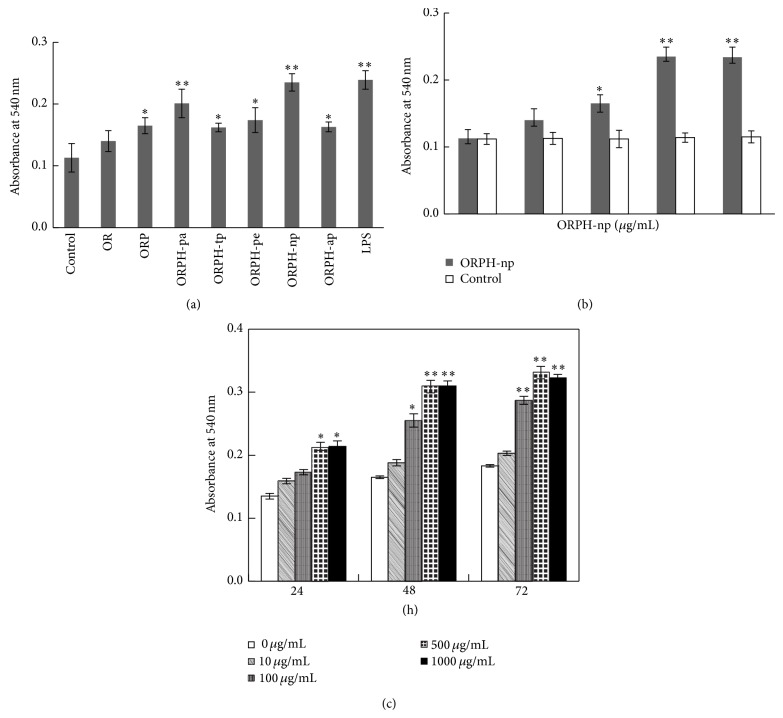
Effect of protein hydrolysate of Oviductus Ranae on phagocytosis. (a) After treatment with test substances (500 *μ*g/mL) or LPS 1 *μ*g/mL for 48 h, RAW 264.7 cells were used to test the phagocytosis by neutral red uptake assay. Control, untreated cells. (b) RAW 264.7 cells were pretreated with ORPH-np (0, 10, 100, 500, and 1000 *μ*g/mL) for 48 h and were used to test the phagocytosis by neutral red uptake assay. Control cells were pretreated with the medium that contained same levels of neutral protease with ORPH-np, the neutral protease was processed by the same procedure with ORPH-np. (c) RAW 264.7 cells were pretreated with ORPH-np (0, 10, 100, 500, and 1000 *μ*g/mL) for 24, 48, and 72 h and were used to test the phagocytosis by neutral red uptake assay. Control, 0 *μ*g/mL. Results were expressed as means ± S.D. of four separate experiments. Statistical significance test for comparison with untreated group was done by *t*-test. ^*^ *P* < 0.05; ^**^ *P* < 0.01.

To determine the effect of Oviductus Ranae protein and protein hydrolysates on the phagocytic activity of macrophage, the neutral red uptake assay was performed. After incubation in culture medium with or without test substances (500 *μ*g/mL) for 48 h, RAW 264.7 macrophages cells were used to test the phagocytosis. Interesting, compared with the control (without test substances), Oviductus Ranae protein and all hydrolysates enhanced the phagocytic activity of macrophage (Figure 2(a)). Among them, ORPH-np exhibited the most significant promoting activity (macrophage phagocytosis was increased by 109.1% compared with untreated group, *P* < 0.01), which is as high as the LPS-treated positive control group (Figure 2(a)). To eliminate the effect of neutral protease on phagocytic activity of macrophage, the cells pretreated with the medium contained same levels of neutral protease with ORPH-np were as control, the effect of ORPH-np (10–1000 *μ*g/mL) on phagocytic activity of macrophage was investigated. The result indicated that ORPH-np promoted the phagocytosis of macrophage, and the activity revealed in a dose-dependent manner in the concentration range of 10–500 *μ*g/mL and did not result from neutral protease in ORPH-np (Figure 2(b)). However, this manner was not continued at the concentration of 1000 *μ*g/mL. Meanwhile, we also asked whether the effect of ORPH-np was in a time-dependent manner. The data shown in Figure 2(c) suggested that the phagocytic activity of macrophage was increased in a time-dependent manner in the time course of 0–48 h, yet prolonged treatment (72 h) could not significantly increase the phagocytic activity of macrophage. These results showed that ORPH-np was able to regulate significantly phagocytosis of macrophage in a dose and time-dependent manner.

### 3.3. Effect of Protein Hydrolysate of Oviductus Ranae on NO Production

**Figure 3:**
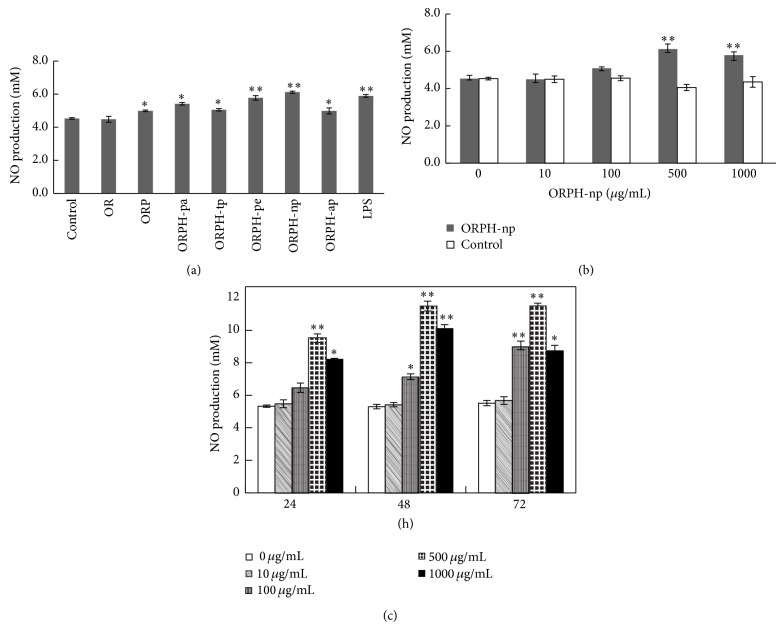
Effect of protein hydrolysate of Oviductus Ranae on NO production. (a) RAW 264.7 cells were pretreated with test substances (500 *μ*g/mL) or LPS (1 *μ*g/mL) for 48 h. The supernatant nitrite levels were determined using Griess reagent. Control, untreated. (b) RAW 264.7 cells were pretreated with ORPH-np (0, 10, 100, 500, and 1000 *μ*g/mL) for 48 h, the supernatant nitrite levels were determined using Griess reagent. Control cells were pretreated with the medium that contained same levels of neutral protease with ORPH-np, the neutral protease was processed by the same procedure with ORPH. (c) RAW 264.7 cells were pretreated with ORPH-np (0, 10, 100, 500, and 1000 *μ*g/mL) for 24, 48, and 72 h and the supernatant nitrite levels were determined using Griess reagent. Control, 0 *μ*g/mL. Results were expressed as means ± S.D. of four separate experiments. Statistical significance test for comparison with untreated group was done by *t*-test. ^*^ *P* < 0.05; ^**^ *P* < 0.01.

To further investigate whether ORPH-np activates macrophages, NO production of RAW 264.7 cells were determined by Griess assay. After cells were incubated with Oviductus Ranae protein or protein hydrolysates (500 *μ*g/mL) for 24 h, NO level of all experimental groups were significantly elevated compared to the control group. The highest induction was observed in the ORPH-np group, in which NO concentration was increased by 34.9% (*P* < 0.01) (Figure 3(a)). The stimulatory activity of ORPH-np also revealed a dose-dependent fashion in the concentration range of 10–500 *μ*g/mL (stopped at 1000 *μ*g/mL.) and did not result from neutral protease in ORPH-np (Figure 3(b)). Similarly, we also examined whether there might be a time-dependent manner in terms of the effect of ORPH-np. Again, as shown in Figure 3(c), NO production of macrophage was increased in a time-dependent manner from 0 to 48 h, while no further change was seen with extended treatment (from 48–72 h). The above results demonstrated that ORPH-np regulated significantly phagocytosis and NO production of macrophage in a dose and time-dependent manner. From now on in the study, ORPH represents ORPH-np unless specified.

### 3.4. Effect of ORPH on the mRNA and Protein Expression of iNOS

**Figure 4:**
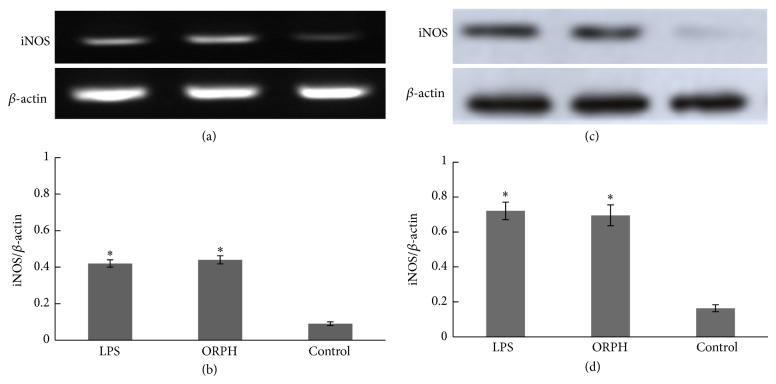
Effect of ORPH on expression of iNOS in RAW 264.7 cells. (a) Cells were incubated with ORPH (500 *μ*g/mL) or LPS (1 *μ*g/mL) for 24 h. Total RNA was prepared and mRNA levels encoding iNOS was measured by RT-PCR. *β*-actin was used as an internal control. (b) Corresponding quantification data of iNOS mRNA expressional levels. (c) The protein level of iNOS in ORPH-stimulated RAW 264.7 cells was analyzed by Western blotting. (d) Corresponding quantification data of iNOS protein levels. ORPH, Oviductus Ranae protein hydrolysate prepared with neutral protease. Control, untreated. The results were stated in iNOS versus *β*-actin; data are expressed as mean ± SD (*n* = 3). Statistical significance test for comparison with untreated group was done by *t*-test. ^*^ *P* < 0.05; ^**^ *P* < 0.01.

NO expressions are highly regulated by the synthesis of iNOS [19, 20]. Therefore, the effect of ORPH on the expression levels of iNOS mRNA and protein in RAW 264.7 cells were investigated by RT-PCR and Western blot, and the results were showed in Figures 4(a) and 4(c). Both the expression levels of iNOS mRNA and protein increased remarkably after incubation with ORPH for 24 h (*P* < 0.05), while *β*-actin remained unchanged (Figures 4(b) and 4(d)). These results showed that ORPH did increase the mRNA and protein expression of iNOS in RAW 264.7 cells.

### 3.5. Effect of ORPH on Macrophage-Related Cytokine Production

**Figure 5:**
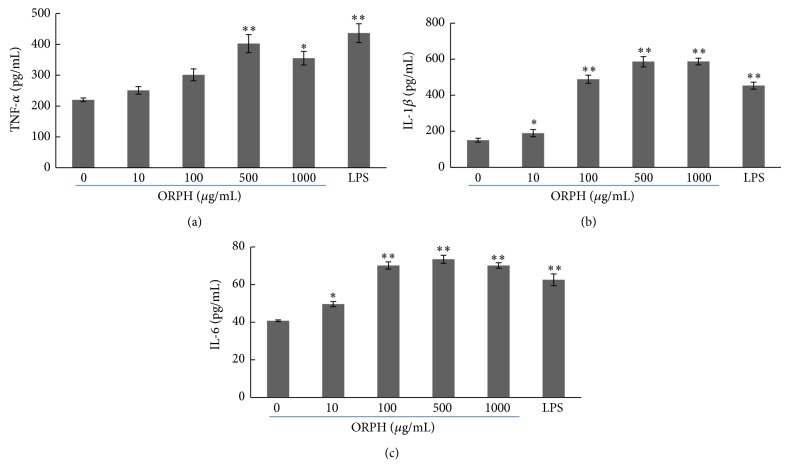
Effects of ORPH on macrophage-related cytokine production in macrophages. RAW 264.7 cells (3 × 10^5^ cells/well) were cultured for 24 h in the presence of media with ORPH (0–1000 *μ*g/mL) or LPS (1 *μ*g/mL). The amounts of TNF-*α* (a), IL-1*β* (b), and IL-6 (c) released into the culture media were measured by immunoassays. ORPH, Oviductus Ranae protein hydrolysate prepared with neutral protease. Results were expressed as means ± S.D. of four separate experiments. Statistical significance test for comparison with untreated group was done by *t*-test. ^*^ *P* < 0.05; ^**^ *P* < 0.01.

To assess the effects of ORPH on TNF-*α*, IL-1*β*, and IL-6 production by activated macrophages, RAW 264.7 cells were incubated in culture medium in the presence of ORPH, and the quantities of these cytokines secreted into the culture supernatants were monitored by ELISA. AS shown as in Figure 5, a dose-dependent manner of TNF-*α*, IL-1*β*, and IL-6 production was observed again, when the cells were treated by a titration of ORPH (10–500 *μ*g/mL). After cells were incubated with 100 or 500 *μ*g/mL of ORPH, marked increase was detected in TNF-*α*, IL-1*β*, and IL-6 secretion in the supernatant. 100 *μ*g/mL of ORPH was able to increase the production of TNF-*α* by 37%, IL-1*β* by 225%, and IL-6 by 75% whereas 500 *μ*g/mL of ORPH enhanced production of TNF-*α* by 83%, IL-1*β* by 290%, and IL-6 by 85% compared to the medium control (untreated group, 0 mg/mL). However, this dose-dependent manner ceased at the concentration 1000 *μ*g/mL. These results indicated that ORPH exhibited significant immunostimulatory activity on macrophage related cytokine production.

### 3.6. Effect of Peptide with Different Molecular Weights on NO and TNF-*α* Production

**Figure 6:**
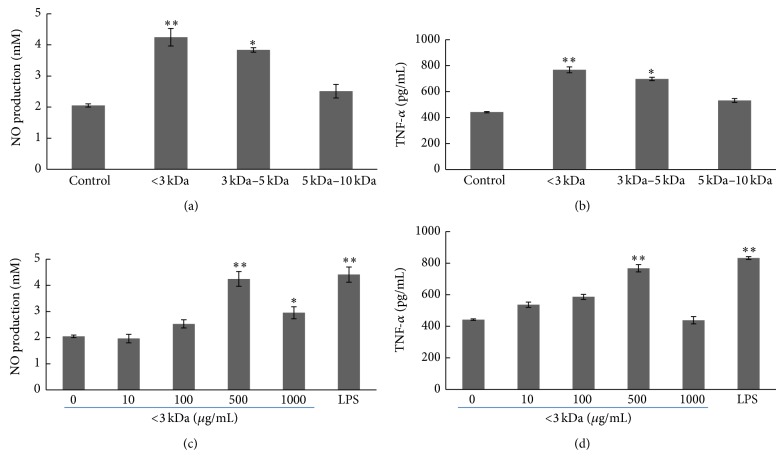
Effect of the peptide with different molecular weights on the NO and TNF-*α* production of RAW 264.7 cells. (a) and (b) cells were pretreated with or without test substances (500 *μ*g/mL), respectively. The supernatant nitrite levels and TNF-*α* were determined using Griess reagent and ELISA kit, respectively. Control, 0 *μ*g/mL. (c) and (d) cells were cultured in the presence of media with the <3 kDa fraction (0–1000 *μ*g/mL) or LPS (1 *μ*g/mL). The amounts of NO and TNF-*α* in the supernatant were determined using Griess reagent and ELISA kit, respectively. ORPH, Oviductus Ranae protein hydrolysate prepared with neutral protease. Results were expressed as means ± S.D. of four separate experiments. Statistical significance test for comparison with untreated group was done by *t*-test. ^*^ *P* < 0.05; ^**^ *P* < 0.01.

The peptide length and molecular weight distribution of hydrolysates are considered to be closely related to their biological activities [21, 22]. In order to study the effect of molecular weight on the immunomodulatory activity, ORPH was separated into four different fractions by ultrafiltration. The effects of fractions with different molecular weights on NO and TNF-*α* production of macrophages were investigated at 500 *μ*g/mL level (Figures 6(a) and 6(b)). The <3 kDa and 3–5 kDa fractions enhanced activity significantly, and <3 kDa peptide showed the highest stimulatory activity. The peptide of <3 kDa revealed stimulatory activity in a dose-dependent manner in the concentration range of 10–500 *μ*g/mL and showed downregulatory trend at 1000 *μ*g/mL (Figures 6(c) and 6(d)). The modulatory manner of the peptide with <3 kDa molecular weight was the same as ORPH. Several reports have shown that relatively low molecular weight peptide exhibited immunomodulating activity [23, 24]. Based on these results, the peptide with <3 kDa molecular weight was the fraction responsible for the immunomodulatory activity.

### 3.7. Effect of ORPH on the Activation of NF-*κ*B

**Figure 7:**
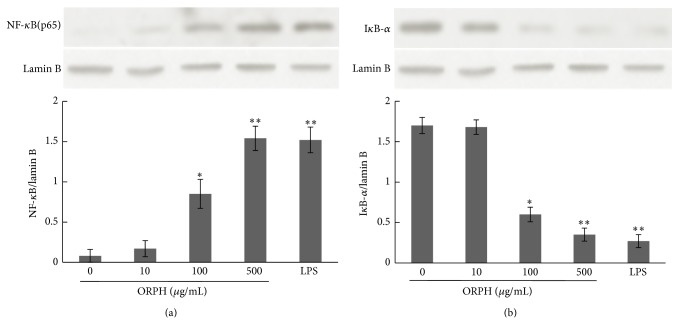
Effect of ORPH on nuclear NF-*κ*B. (a) and cytoplasmic I*κ*B-*α* (b) expression level by RAW 264.7 cells. Cells were cultured for 24 h in the presence of media with ORPH (0–500 *μ*g/mL) or LPS (1 *μ*g/mL). Western blotting was performed to detect the protein level of nuclear NF-*κ*B and cytoplasmic I*κ*B-*α*. The levels of lamin B were measured as internal loading controls. The results were analyzed by iPP 6.0 software and stated in NF-jB versus Lamin B, data are expressed as mean ± S.D. (*n* = 3). Statistical significance test for comparison with untreated group was done by *t*-test. ^*^ *P* < 0.05; ^**^ *P* < 0.01.

Given that ORPH showed immunostimulatory activity on macrophage and NF-*κ*B is one of the most important transcription factors in regulation of macrophage activation, the content of nuclear NF-*κ*B and cytoplasmic I*κ*B-*α* was therefore detected by western blot. After incubating RAW 264.7 with ORPH (0, 10, 100, and 500 *μ*g/mL) or LPS (1 *μ*g/mL) for 24 h, cytoplasmic and nuclear protein were extracted by the Cytoplasmic and Nuclear protein extract kit. The content of nuclear NF-*κ*B gradually increased after treated with ORPH (Figure 7(a)), while that of cytoplasmic I*κ*B-*α* displayed an opposite trend in a concentration-dependent manner (Figure 7(b)). The results showed that the effect of ORPH on macrophage activities was likely to be mediated by the I*κ*B-NF-*κ*B signaling pathway.

## 4. Discussion

To our knowledge, this is the first study that describes immunostimulatory activity of ORPH on macrophages* in vitro*. In this study, phagocytic assay by neutral red uptake illustrated that ORPH stimulated the phagocytosis of macrophages. The nitrite levels in the culture supernatant determined using Griess reagent revealed the elevation of NO production after treatment with ORPH. RT-PCR and Western blotting assay indicated that ORPH promoted the mRNA and protein expression of iNOS. ELISA assay showed the elevation of production of TNF-*α*, IL-1*β*, and IL-6 after treatment with ORPH. Furthermore, the results of Western blotting demonstrated that NF-*κ*B levels in nucleuses increased after ORPH treatment. These findings suggested that effects of ORPH on macrophage activity might be due to the activation of NF-*κ*B pathway.

After phagocytic uptake, macrophages turn their role into antigen-presenting cells with expression of higher levels of costimulatory molecules and then mediate an interaction between T cells and macrophages [25]. In this study, phagocytosis assay of neutral red uptake internalization showed that ORPH significantly increased the phagocytosis of RAW 264.7 cells, which suggested that ORPH treatment might result in the initiation of immune reaction against foreign materials. Previous studies have shown that similar immunomodulating effects were found in enzymatic protein hydrolysates of whey [26, 27], fish [24, 28], and oyster [29]. In oyster, an increased phagocytic activity was detected by oyster protein hydrolysates treatment [29]. In pacific whiting (Merluccius productus), phagocytic activity of macrophages was enhanced following the fish protein concentrate (FPC) treatment suggesting that the FPC induced the release of cytokines able to activate immune cells for induction of the immune response [24]. In milk, the peptide from alpha-lactalbumin stimulated, in a dose-dependent manner, the binding of human senescent red blood cells to macrophage cells and phagocytosis by these cells [30]. In the present study, ORPH, as another valuable source of biologically active peptides, was shown to closely relate to this similar immunostimulatory effect as well.

During the phagocytic process, activated macrophages produce NO. Since NO is related to the cytolytic function of macrophages against a variety of pathogens, increased NO synthesis can induce immunostimulatory activity in macrophages. When activated, macrophages also inhibit the invasion of microorganisms by releasing cytokines. NO is involved in diverse functions, including nerve growth, wound healing, and the immune response [9]. The present results showed that ORPH treatment increased the nitrite concentration in the supernatants in a dose and time dependent manner. To confirm the increase of NO production, the gene and protein expressions of iNOS were also analyzed by RT-PCR and western blotting in RAW 264.7 cells. Consistent with nitrite concentration data, the results collectively indicated that ORPH elevated the mRNA and protein level of iNOS. In the meantime, macrophage-related cytokines TNF-*α*, IL-1*β*, and IL-6 were also investigated in this study to confirm that ORPH is an immunostimulator. Our results showed that the secretion levels of TNF-*α*, IL-1*β*, and IL-6 were upregulated in ORPH-treated macrophages, suggesting the possible roles of macrophages in the antitumor activity upon ORPH treatment, as previously demonstrated for fermented milk [31] and fish protein hydrolysates [28]. Macrophages stimulated by ORPH produced TNF-*α* and IL-1*β*, meaning that ORPH could induce the production of cytokines, resulting in the enhancement of TNF-*α* cytostaticity. IL-6 is mainly produced by antigen-presenting cells, and has multiple biological activities in various cell types. In the development of cell-mediated immune responses, IL-6 synergizes with IL-1*β* and promotes T cell proliferation, T helper cell differentiation, and development of T cell-mediated cytotoxicity by CD81 cells [32, 33]. Therefore, ORPH stimulates macrophage-derived TNF-*α*, IL-1*β*, and IL-6 production, implying that ORPH might induce Th immune responses. The activation of macrophages through PRR receptors induces the expression of major histocompatibility complex (MHC) class II and costimulatory molecules such as CD40, CD80, and CD86, which are critical for T cell activation [34]. To further confirm that protein hydrolysate of Oviductus Ranae is effective for immunostimulation, future studies are needed to clarify the effect of protein hydrolysates on the expression of activation markers such as MHC class II, CD80, and CD86 induced by LPS in macrophages.

NF-*κ*B plays a primary role in the transcriptional regulation of various genes such as macrophage-related cytokines [35]. When signals for the activation of NF-*κ*B are received, serine residues in I*κ*B are phosphorylated and dissociated from NF-*κ*B, which is then transferred to the nucleus as an activated transcription factor [36]. After the degradation of I*κ*B, the binding sites of p50–p65 dimer are then exposed to combine with the *κ*B motif. Then, the NF-*κ*B p65 subunit would transfer from the cytoplasm to the nucleus with potent activity. In its active DNA-binding form, a large number of genes such as iNOS, IL-12p40, IL-1*β*, TNF-*α*, and IL-6 are regulated [17]. The present study revealed that ORPH increased the production of NO and the macrophage-related cytokines TNF-*α*, IL-1*β*, and IL-6. ORPH increased the level of NF-*κ*B activation in macrophages, which implicated that ORPH might stimulate the release of NF-*κ*B from I*κ*B-*α* and transfer from cytoplasm to nucleus. Therefore, the present results raised the possibility that ORPH might induce macrophage activation through the NF-*κ*B signaling pathway. It is well established that macrophages play pivotal roles in both innate and adaptive immune response to pathogens, including phagocytosis, antigen presentation, and cytokine secretion [37, 38]. However, large amounts of macrophage-derived inflammatory mediators could also lead to the inflammatory responses. In particular, excessive production of TNF-*α*, IL-1*β*, and IL-6 could aggravate inflammation diseases [39, 40]. In addition, increased expression of iNOS and release of large amounts of NO also play a significant part in the pathogenesis of inflammatory and other diseases such as rheumatoid arthritis, chronic hepatitis, and pulmonary fibrosis [41, 42]. The present results showed that extended the test ORPH concentration 1000 *μ*g/mL and found that production of TNF-*α*, IL-1*β*, IL-6, and NO was upregulated in a dose-dependent manner in the concentration range of 10–500 *μ*g/mL, and the trend ceased at the concentration 1000 *μ*g/mL. These results suggested that proper regulation of these macrophage functions by immunomodulatory molecules such as ORPH could help a host to protect itself from various pathologic or cancerous attacks.

In conclusion, the present results demonstrated that ORPH was able to not only stimulate murine macrophage RAW 264.7 to secrete inflammatory cytokines, but also increase their phagocytic activity and NO production. Also, the increase in nuclear NF-*κ*B level upon ORPH treatment was confirmed. These findings suggested that ORPH could induce macrophage activation, and this immunostimulatory activity was highly likely to be associated with the activation of NF-*κ*B signaling pathway.

## Figures and Tables

**Table 1:** Conditions of enzymatic hydrolysis.

Enzyme	pH	Temperature (°C)
Papain	7.0	60
Trypsin	8.0	40
Neutral protease	7.5	55
Pepsin	2.5	37
Alkaline protease	9.0	50
